# Mania a Potu

**Published:** 1875-11

**Authors:** L. A. Dugas

**Affiliations:** Augusta


### MANIA A POTU.

BY L. A. DUGAS, M.D., OF AUGUSTA.

*Read before the Medical Association of Georgia, in April*, 1875.

Having been forcibly impressed, during my pupilage, with the fatality then attending mania 'a potu, as treated in the hospitals of our large Northern cities, I determined, upon the commencement of my professional career, to adopt a different plan of treatment, and to carefully note the results. At that time practitioners usually relied principally upon opiates, in one form or another, to induce sleep, and upon calomel to correct the secretions, both of which were considered necessary to success. The opiates were often administered in enormous quantities, and the cases which did not terminate fatally, were not unfrequently terribly salivated, making convalescence both protracted and precarious. The violence of the delirium was made so great as to require the patients to be confined in the straight jacket, then in common use in other forms of mania.

I now feel it a duty to lay before the profession the result of my long experience in a more formal manner than that of clinical instructions, made to the classes of the Medical College of Georgia for upwards of forty years.

My plan of treatment is very simple. It consists of the administration, once or twice a day, of cold water affusions, of such food as may be relished, and sometimes of two or three small drinks of toddy per day. If the patient is very unruly, one or two intelligent persons should attend him day and night as long as it may be necessary.

For the affusions a large tub should be provided, if a bathing tub be not convenient, three or four buckets of water, with a pitcher and napkins placed near the tub, and one or two assistants. The patient should then be stripped of all garments and seated in the tub, the aid's hands should be placed upon the patient's shoulders, so as to prevent his jumping up, and the physician should then pour one pitcherful after another over the patient's head, in a regular stream, which will pass down over the entire body. The affusion is to be continued from one to three minutes, *by the watch.* I have never seen one who needed or could bear it over three minutes. However wild or violent the patient may be, he soon becomes quiet, begs most pitiously to be released, and makes the most solemn promises to be quiet thereafter. The pulse, which was full and strong, soon looses the peculiarities, and becomes small and frequent. If the affusion be continued too long the pulse may become almost imperceptible. The physician should therefore watch this carefully, and cease whenever the patient is entirely rational, and the pulse sufficiently subdued.

Let the aid then rub the patient rapidly, and with force until the skin is dry, and red with reaction. Place him then promptly in bed, cover him warmly, and tell him to go to sleep. The room should be darkened, and a nurse left near him. In most cases the patient will be asleep in a few minutes, and continue so more or less long, according to his previous loss of rest. He will sometimes sleep six or eight hours, and awake perfectly rational. He should then be fed, and allowed a little toddy, if he craves it.

If this affusion be administered in the morning, and the relief from delirium be not complete at night, it should then be repeated; but I am in the habit of repeating it every morning until the perfect restoration, which may vary from two to six days. Too much attention cannot be given to the taking of food, by complying with the caprices of his appetite or taste.

The affusion should always be done in presence of the physician, as very few nurses can be trusted for its proper administration and timely suspension. If the bowels are too slow, an enema of cold water will soon empty the rectum. I never allow the use of laxatives, and still less of cathartics, lest unnecessary purging be caused. The fact is that patients have usually eaten so little during the attack that there can rarely be any injurious accumulation in the intestines. Diarrhœa must be immediately checked by ordinary astringents, avoiding opium or camphor. During the whole management of the case, I eschew all narcotics, with the exception of the toddy as above stated.

We know that while some cases of this disease occur *during* the excessive use of alcoholics, others come on *after* the cessation of these potations. Hence it is that we should discriminate between them, and advise toddy only for those who seem to suffer from its absence. The other class of cases do better without it.

Now, as to the success of this plan of treatment, I have to say that, with one exception, I have never lost a case when called to see it before it was too late to expect good from any treatment, and these have been very few. In the exceptional case the patient had old complications, which would have rendered any treatment unsuccessful.

